# In-situ synthesis and integration of gold nanoparticles into 3D printed optical fiber probes

**DOI:** 10.1038/s41598-024-81139-x

**Published:** 2024-11-29

**Authors:** Dileep Chekkaramkodi, Said El Turk, Murad Ali, Haider Butt

**Affiliations:** Department of Mechanical & Nuclear Engineering, Khalifa University of Science and Technology, Abu Dhabi, UAE

**Keywords:** Optical probes, Gold nanoparticles, Additive manufacturing, Polymer nanocomposite, 3D printing, Materials science, Nanoscience and technology, Optics and photonics

## Abstract

This work uses the polymeric reduction method to explore the in-situ synthesis of gold nanoparticles (AuNPs) within 3D-printed optical fiber probes (OFPs). Digital light processing (DLP) 3D printing is employed to fabricate the OFPs using a resin consisting of hydroxyethyl methacrylate (HEMA) and polyethylene glycol diacrylate (PEGDA). After printing, OFPs were immersed in a boiling gold precursor solution to facilitate the synthesis of AuNPs inside the polymer matrix. We produced single material (HEMA/PEGDA) and multimaterial (HEMA/PEGDA + Dentaclear) OFPs loaded with AuNPs at different concentrations. Scanning electron microscopy analysis confirmed the effective distribution and dispersion of AuNPs within the polymer matrix. The optical properties, including reflection and transmission spectra, are comprehensively measured using customized setups. The localized surface plasmon resonance of the embedded AuNPs created a distinct dip in the 500–600 nm wavelength range. Higher AuNP concentrations and longer dipping times enhanced light absorption, reducing reflection and transmission intensities. Multimaterial OFPs also exhibited tunable wavelength filtering capabilities based on the AuNP concentration. The AuNP-loaded OFPs demonstrated stable optical performance across varying temperatures and pH environments, highlighting their potential for diverse applications.

## Introduction

Gold nanoparticles (AuNPs) have distinct and intriguing optical features due to a phenomenon known as surface plasmon resonance (SPR)^1–4^. Surface plasmons are oscillations of free electrons at the interface of a metal and a dielectric substance. When light interacts with AuNPs, its fluctuating electromagnetic field resonates with free electrons in the conduction band^5,6^. This causes free electrons to oscillate collectively, resulting in a SPR. At the SPR frequency, AuNPs strongly absorb and scatter light, producing bright colors. This explains why colloidal AuNP solutions appear red or purple, depending on the size and form of the nanoparticles. In addition, if the diameter of the nanoparticles is comparable to the wavelength of light, resulting in a phenomenon called localized surface plasmon resonance (LSPR)^7,8^. This results in significant increases in the electromagnetic field at the nanoparticle surface. AuNP’s SPR properties greatly rely on their size, shape, and surrounding environment. By adjusting these factors, it is possible to tailor the SPR wavelength and intensity to various applications in optics, sensing, imaging, catalysis, and biomedical research^9–11^.

In recent years, researchers have investigated the insertion of AuNPs into diverse polymer matrices to produce nanocomposite materials with specific optical characteristics for various applications^12–14^. Embedding these AuNPs within the optical fibers endows the fibers with plasmonic capabilities. Light passing through optical fibers, especially in areas containing AuNPs, can induce LSPR. So the optical fiber probes not only transmit light but also engage with the LSPR of the AuNPs, resulting in distinct optical phenomena, including increased light absorption or scattering at specific wavelengths. Ismail et al. investigated the incorporation of AuNPs in the Polystyrene (PS) matrix for possible applications in optoelectronics and sensing^15^. This study uses PS microspheres doped with methyl orange and coated with AuNPs. They analyzed the optical and structural characteristics of the porous films before and after the inclusion of Au nanoparticles. Following the introduction of AuNPs, the optical energy gap grew from 2 to 3.5 eV. They conducted a study on the electrical and photoresponse properties of Au-PS/p-Si heterojunctions. The results demonstrated enhanced rectification characteristics and two distinct response peaks at 450 and 900 nm wavelengths.

Muranaka et al. investigated the synthesis of gold nanoparticles within a hydrogel using multiphoton photoreduction for plasmonic sensing^16^. The utilization of metal nanoparticles for plasmonic sensing has garnered significant interest due to their exceptional sensitivity to detection. This study involved the synthesis of AuNPs using femtosecond laser pulses within a hydrogel that contained gold chloride and glutamine. Glutamine inhibited AuNP development in the area beyond the laser focus point. By using the suggested method to make AuNPs, structures with optical properties linked to localized surface plasmon resonance can be precisely produced. They used hydrogels with structures to identify the common biomolecules found in sweat. Among all the biomolecules, only urea caused a blue shift in the absorbance spectra peaks of the AuNPs inside hydrogels. The findings suggest that hydrogels with engineered architectures can effectively detect the presence of urea.

The introduction of AuNPs into polymer matrices has numerous benefits for optical applications^17,18^. The polymer matrix protects the AuNPs by increasing their stability and avoiding aggregation, which can deteriorate their optical capabilities. It is also possible to modify the polymer matrix to possess specific optical qualities like transparency or refractive index, which combine with the plasmonic capabilities of the AuNPs to achieve the desired functionality^19,20^. Furthermore, the polymer matrix enables fine control over the size, shape, and distribution of AuNPs inside the nanocomposite, optimizing their optical properties for specific applications^21^. This control is critical for producing desired optical effects, such as increased light absorption, scattering, or modifying the plasmonic resonance wavelengths. Even though AuNP-loaded polymers show a lot of promise for optical uses, it is still hard to make sure that the nanoparticles are evenly distributed within the polymer matrix, to control the size and shape of the nanoparticles while they are being produced, and to figure out how stable these nanocomposites are in the polymer matrix.

To fabricate optical fibers, different techniques are used, including Outside Vapour Deposition, Modified Chemical Vapour Deposition, Vapour Axial Deposition, Rod-in-Tube, Stack-and-Draw, and Plasma Chemical Vapour Deposition^22,23^. These approaches provide distinct advantages in fiber composition regulation, manufacturing efficiency, and particular optical qualities, enabling manufacturers to customize the production process to achieve their preferred attributes and applications. 3D printing, or additive manufacturing (AM), is a relatively new manufacturing method used to fabricate optical components, that involves the gradual addition of material layers to construct a three-dimensional object^24–28^. 3D printing has various benefits in photonics, including fabricating intricate structures; expedited prototyping; cost-effectiveness; material variety; integration and miniaturization; on-demand production; and design flexibility^29^. It facilitates the fabrication of customized optical components, such as lenses, waveguides, and diffraction gratings with exceptional precision. Rapid prototyping diminishes development duration and expenses, rendering it optimal for research and development environments. Multi-material 3D printing is the process of printing two or more different materials together, which allows for the incorporation of various materials and qualities throughout the object’s shape^30^. This method is essential for specific applications, such as biomimetic components, tissue engineering, robotic parts, and optical equipment^31,32^. Fahad et al. fabricated optical fiber sensors made of polymers using micro-stereolithography 3D printing technique^33^. He added thermochromic powder as a sensing element in the polymer matrix to sense the change in temperature by analyzing reflection measurements. Utilizing multi-material 3D printing can greatly decrease manufacturing time and optimize production processes. Methods such as fused deposition modeling (FDM), vat photopolymerization, and material extrusion allow using several materials for 3D printing^34,35^. Digital Light Processing (DLP) is a method based on vat photopolymerization that uses targeted two-dimensional patterns of ultraviolet (UV) light to solidify the material in the vat^36^. DLP has gained recognition for its exceptional resolution, hydrogel printing capacity, and superior optical component printing performance. The tight and continuous adhesion between many printed layers minimizes optical transmission losses at the interfaces between the layers.

This study presents a new method for synthesizing AuNPs within 3D printed optical fiber probes using the polymeric reduction method. The process involves immersing 3D printed optical fiber probes (OFPs) made of hydroxyethyl methacrylate (HEMA) based resin in a boiling solution of gold precursor for a specific duration, allowing AuNPs to be incorporated directly and selectively only within the hydrogel matrix. Multi-material optical fibers were printed and selective incorporation of gold nanoparticles into the preordained printed segments was achieved. These OFPs were tested for their optical properties and the loaded AuNPs were characterized using scanning electron microscopy (SEM), Fourier transform infrared microscopy (FTIR), and transmission electron microscopy (TEM). Customized experimental setups are used to evaluate the probes’ optical performance in both transmission and reflection modes.

## Materials and methods

**Fig. 1:**
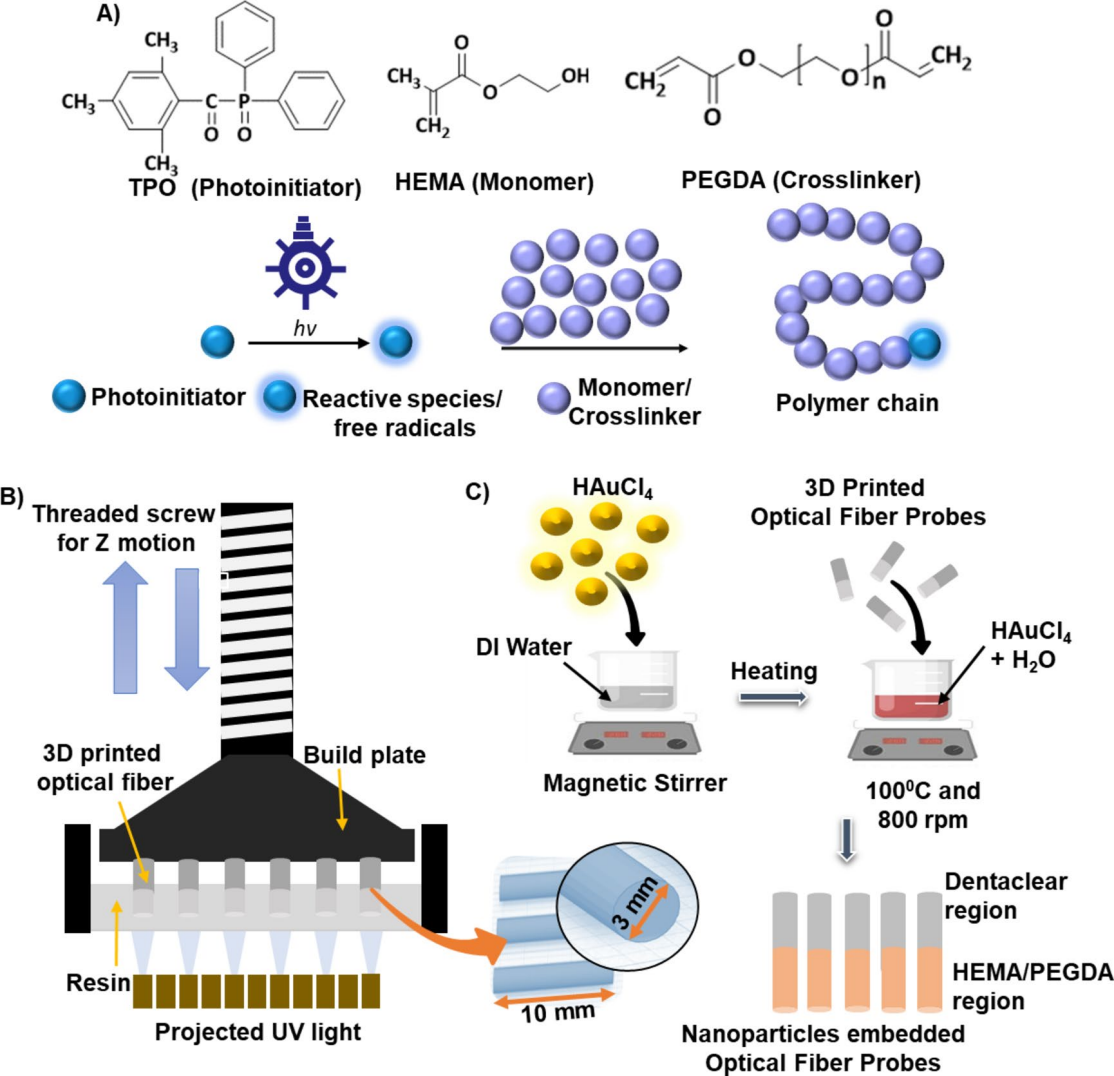
(**A**) The chemical reaction under the influence of UV light in TPO and the cross-linking of HEMA and PEGDA, (**B**) 3D printing of the optical fiber probes, (**C**) Integrating gold nanoparticles in the optical fiber probes. Gold nanoparticles are only synthesized within the region printed with HEMA resin.

The initial raw materials used for printing OFPs were hydroxymethyl methacrylate (HEMA, ≥ 99% purity, Sigma-Aldrich, molecular wt. 2200) and polyethylene glycol diacrylate (PEGDA, ≥ 99% purity, Sigma-Aldrich, molecular wt. 2000). Trimethoxylbenzoyl phosphine oxide (TPO, ≥ 99% purity, Sigma-Aldrich) was used as a photoinitiator. Gold (III) chloride hydrate (Au_2_Cl_6_.H_2_O, ≥ 99% purity, Sigma-Aldrich) serves as a precursor for gold nanoparticles. The polymer resin used for 3D printing is formulated by combining HEMA and PEGDA in a weight ratio of 97:3. A 3 wt% TPO was added as a photoinitiator. Then, agitate the mixture using a magnetic stirrer at 500 rpm for 30 min. Figure 1.A shows the chemical reaction initiated by a photoinitiator (TPO) under UV light, which leads to the polymerization of a monomer (HEMA) and a crosslinker (PEGDA), forming a polymer chain. To print multimaterial optical fiber an additional transparent resin Dentaclear (ASIGA, Alexandria, NSW, Australia) was used. The resin mixture comprises methacrylate and diphenyl (2, 4, 6-trimethyl benzoyl) phosphine oxide.

The resin is used to print the OFPs in a DLP 3D printer, Anycubic Photon Mono 4 K, with a resolution of 3840 × 2400px. The 3D printer features a 6.23-inch exposure screen and can print objects with maximum dimensions of 165 × 132 × 80 mm (HWD). This printer uses a UV light source with a wavelength of 405 nm, which falls within the range of the activation wavelengths of the TPO. The normal layer has a thickness of 25 μm and an exposure period of 35 s, while the burn layer has an exposure time of 45 s. The lift speed is 80 mm/min, with a lift distance of 6 mm. A retract speed of 80 mm/min is used.

To print OFPs (1 cm length, 3 mm diameter) with a single material, the prepared HEMA/PEGDA resin was used. For producing multimaterial fiber, a commercially available resin Dentaclear was used to print the first half (5 mm length, 3 mm diameter) of the OFPs, and then the print process was paused to change the vat material to HEMA-PEGDA resin, and subcequently the printing process was restarted. As a result, the multimaterial sample has two parts, Dentaclear and HEMA-PEGDA, each part having a 5 mm length. Figure 1.B depicts the 3D printing process of the OFPs, where a resin is exposed to projected UV light on a build plate, with the motion controlled by a threaded screw.

To prepare the AuNP precursor, 0.1 g and 0.025 g of gold (III) chloride hydrate was dissolved in a beaker containing 60 ml of distilled water. The gold precursor solution was heated to a temperature of 100 °C while being vigorously agitated at 800 rpm. Once the temperature hits 100 °C, the printed OFPs are introduced into the stirring solution. The process of heating and stirring was halted, and the immersed samples were subsequently removed from the solution at intervals of 30 s, 1 min, 2 min, 5 min, and 10 min. Then the OFPs were dried at 50 °C. Four different sets of OFPs were made for this study: pure HEMA-PEGDA + low-concentration gold precursor, pure HEMA-PEGDA + high-concentration gold precursor, multimaterial + low-concentration gold precursor, and multimaterial + high-concentration gold precursor. Figure 1.C illustrates the integration of AuNPs into the OFPs. As observed the gold nanoparticles only get reduced in the regions that are printed by HEMA-PEGDA resin.

The size and shapes of the synthesized AuNPs were investigated using TEM (Tecnai, 200 kV), and the incorporation of AuNPs in the polymer matrix was investigated using SEM (Quanta 250 FEG – ESEM). The functional groups of the polymer were identified using the Spotlight 200 FT-IR Microscopy (Pekin Elmer). The transmission and reflection spectra of the 3D printed OFPs were analyzed using customized measurement setups (UV-Vis spectrophotometer, USB 2000+) along with OceanArt software. The reflection and transmission spectra in the manuscript are averaged from 20 repeated observations in the Ocean ART software, ensuring data reliability and performing the experiments in triplicates. The error percentage is less than 5%.

## Results and discussions

**Fig. 2:**
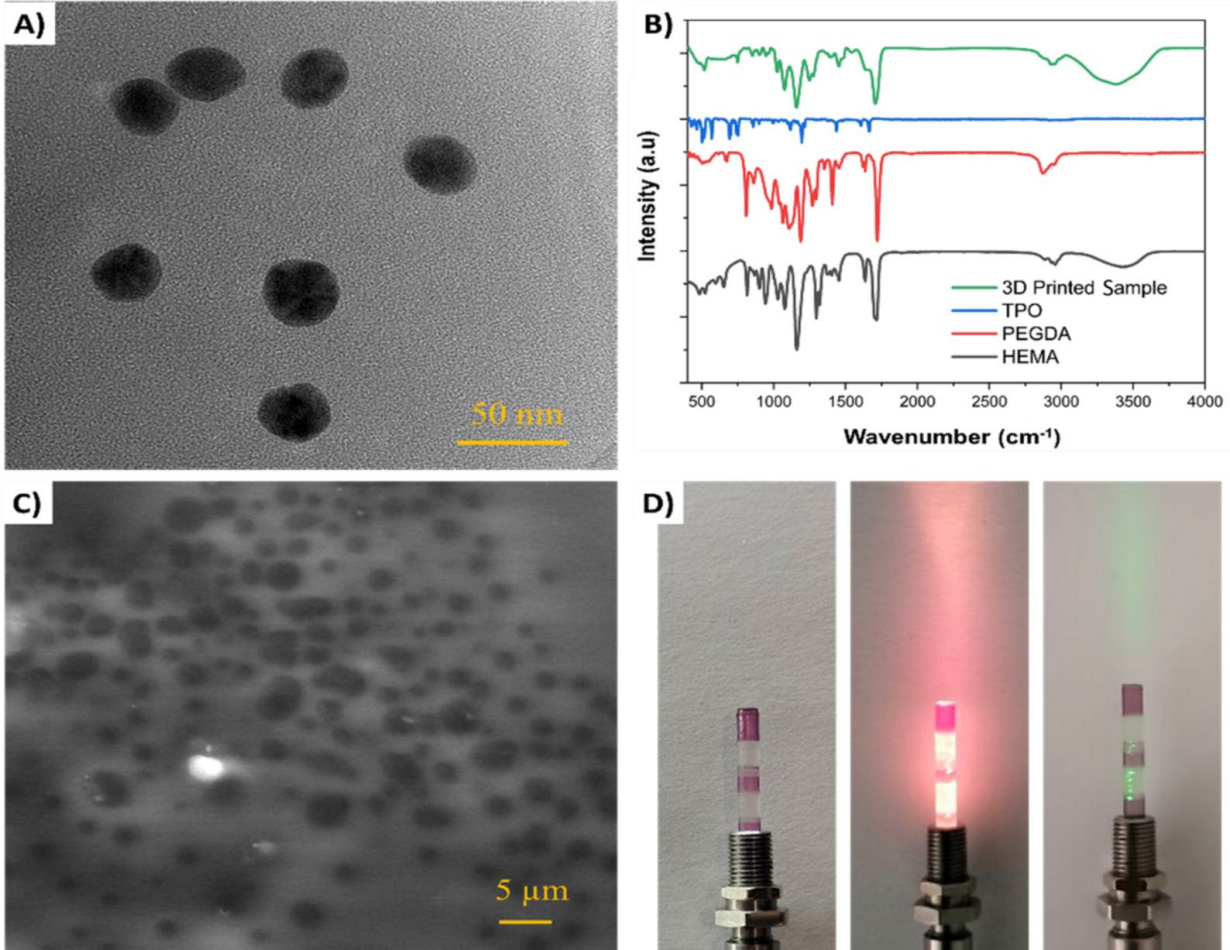
(**A**) TEM image of gold nanoparticles produced by the Turkevich method, (**B**) FTIR spectra of the polymer resin and 3D printed sample, (**C**) SEM images of the nanoparticle embedded OFP by polymer reduction method, (**D**) photographs of fabricated multimaterial OFP under no illumination, illumination under white light, and illumination under a green laser.

Figure 2.A displays a TEM image that reveals the synthesized AuNPs from the AuCl_4_ solution by the Turkevich method, showcasing their spherical shape and size distribution of around 30 nm. Figure 2.B displays the FTIR spectra of HEMA resin, PEGDA resin, TPO, and the 3D printed samples, within the wavenumber range from 400 cm^− 1^ to 4000 cm^− 1^. The peak at 1300 cm^− 1^ corresponds to the C-O bond and the peak at 1660 cm^− 1^ is related to the C = C bond. The peaks at 1720 cm^− 1^, 2900 cm^− 1^, and 3500 cm^− 1^ correspond to the C = O bond, CH_2_ (methylene), and O-H bonds respectively^37–39^. The C = C bonds are critical for reducing gold ions. The FTIR analysis indicates that HEMA and PEGDA hydrogels undergo cross-linking to produce a polymeric resin. Figure 2.C depicts the SEM image of the nanoparticle-embedded OFP by polymeric reduction method at a higher magnification, revealing the distribution and dispersion of the AuNPs within the polymer matrix. From the images, it is clear that the nanoparticles are well-dispersed inside the polymer matrix. The black holes visible in the image are part of the polymer matrix. Figure 2.D showcases photographs of the fabricated multimaterial OFPs under different illumination conditions. The first image shows the OFP under no illumination, while the second image demonstrates its appearance when illuminated with white light. The third image displays the OFP when illuminated with a green laser (wavelength 532 nm). The multimaterial OFP shows clear and AuNP-doped pink-red colored regions corresponding to the regions printed with Dentaclear resin and HEMA-PEGDA resin, respectively.

**Fig. 3:**
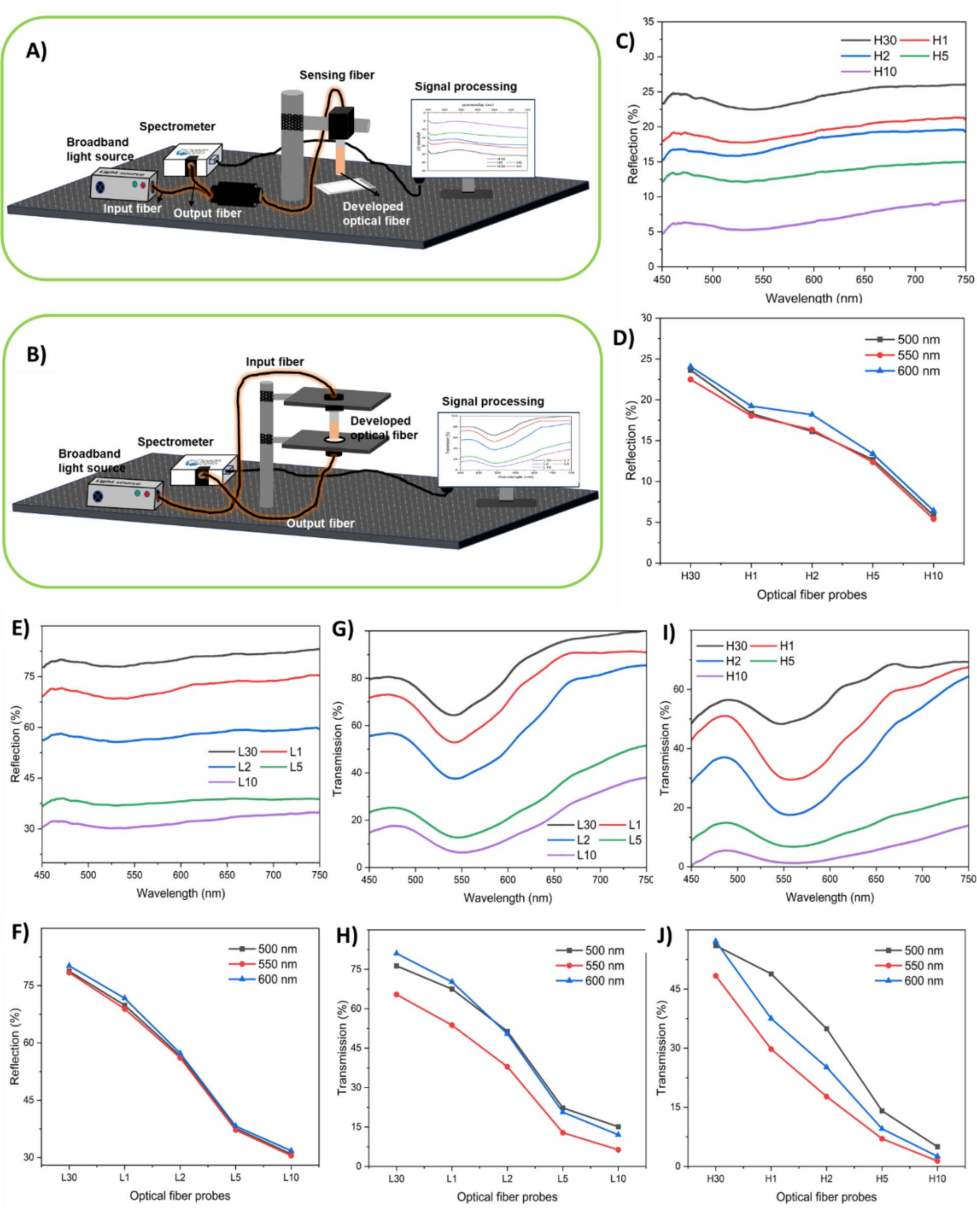
(**A**) Reflection mode measurement setup, (**B**) transmission mode measurement setup, (**C**) reflection spectra from the single material (High-AuNPs) OFPs under white light (the number indicates dipping time 30 s, 1 min, 2 min, 5 min, and 10 min respectively), (**D**) reflection at wavelengths of 500, 550, and 600 nm (High-AuNPs), (**E**) reflection spectra from the single material (low-AuNPs) OFPs under white light, (**F**) reflection at wavelengths 500, 550, and 600 nm (low-AuNPs), (**G**) transmission spectra from the single material (low-AuNPs) OFPs under white light, (**H**) transmission at wavelengths 500, 550, and 600 nm (low-AuNPs), (**I**) transmission spectra from the single material (high-AuNPs) OFPs under white light, and (**J**) transmission at wavelengths 500, 550, and 600 nm (high-AuNPs).

The reflection and transmission of the OFPs are measured using a customized measurement setup shown in Fig. 3.A and 3.B respectively. Figure 3.C illustrates the reflection spectra of OFPs, which are immersed in high-concentration gold solution (H30, H1, H2, H5, H10, where H indicates high concentration solution, the number indicates dipping time 30 s, 1 min, 2 min, 5 min, and 10 min respectively) to integrate the nanoparticles into the OFPs. There is a very slight dip observed in the reflection spectra in the range of 500 nm to 600 nm wavelength, which corresponds to the LSPR of the AuNPs. The reflection from the fibers decreases with an increase in dipping time because more nanoparticle concentrations in the fibers absorb and scatter more light. Figure 3.D depicts the change in reflection intensity of the optical fiber probes at wavelengths 500 nm, 550 nm, and 600 nm. From the graphs, it is identified that the reflection intensity shows a slight dip in the range of 500 to 600 nm. Figure 3.E and 3.F show the reflection spectra of the optical fiber probes immersed in the low-concentration gold solution (L30, L1, L2, L5, L10, where L indicates low concentration solution) and variation in reflection intensity at 500 nm, 550 nm, and 600 nm respectively. A similar trend compared to the optical fiber probes dipped in the high-concentration gold solution is observed. Still, the reflection intensity is higher compared to the previous case because of the low concentration of the embedded nanoparticles in the fiber. Figure 3.G and 3.I depict the transmission spectra of optical fiber probes immersed in low-concentration and higher-concentration gold nanoparticle solutions respectively. A dip in the range of 500 to 600 nm is observed in both cases. Also, the intensity of transmission reduces with an increase in dipping time, the same as in the case of reflection. Figure 3H and J show the variation in transmission percentage at 500 nm, 550 nm, and 600 nm of the optical fiber probes immersed in low-concentration and high-concentration gold solutions respectively.

**Fig. 4:**
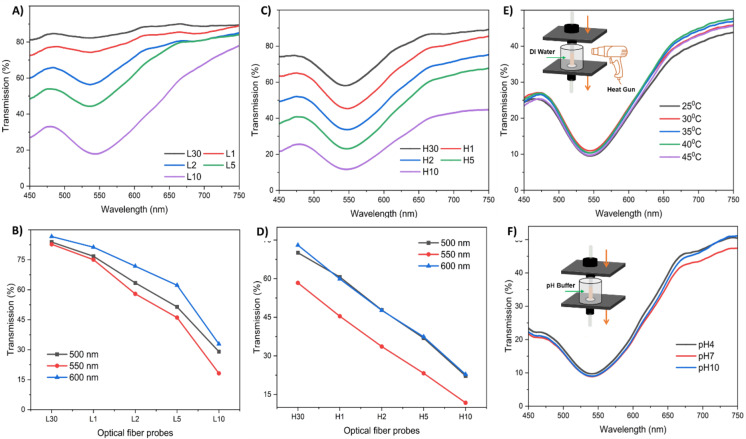
(**A**) Transmission spectra from the multimaterial (low-AuNPs) OFPs under white light, (**B**) transmission at 500, 550, and 600 nm (low-AuNPs), (**C**) transmission spectra from the multimaterial (high-AuNPs) OFPs under white light, (**D**) transmission at 500, 550, and 600 nm (high-AuNPs), (**E**) transmission spectra of multimaterial OFP (L10) at temperatures ranging from 25 °C to 45 °C, and (**F**) transmission spectra of multimaterial OFP (L10) at different pH.

Figure 4 illustrates the transmission results of 3D-printed multimaterial OFPs. Figure 4.A shows the change in transmission spectra from a wavelength of 450 to 750 nm at different dipping times, in low AuNP solution. With dipping time, inserting more AuNPs into the OFPs increases the wavelength filtering to 550 nm. An increase in dipping time reduces the transmission percentage, as in the case of single material OFPs. Figure 4.B shows the transmission through the OFP samples at different wavelengths; 500 nm, 550 nm, and 600 nm, extracted from Fig. 4.A. Figure 4.C illustrates this trend in OFPs immersed in high AuNP solution. Figure 4.D depicts the transmission at wavelengths 500 nm, 550 nm, and 600 nm of OFP, dipped in high AuNP solution. Figure 4.E represents the changes in transmission spectra of OFP (L10) at temperatures ranging from 25 °C to 45 °C. There are no significant changes in the observed temperature variations, indicating that the resin does not undergo much thermal expansion or refractive index changes. Figure 4.F shows the transmission spectra of OFP (L10) at various pH solutions. The transmission change is insignificant with pH. Thus, the transmission of fabricated optical fiber probes is stable in the temperature range of 25 °C to 45 °C and in different pH environments.

**Fig. 5:**
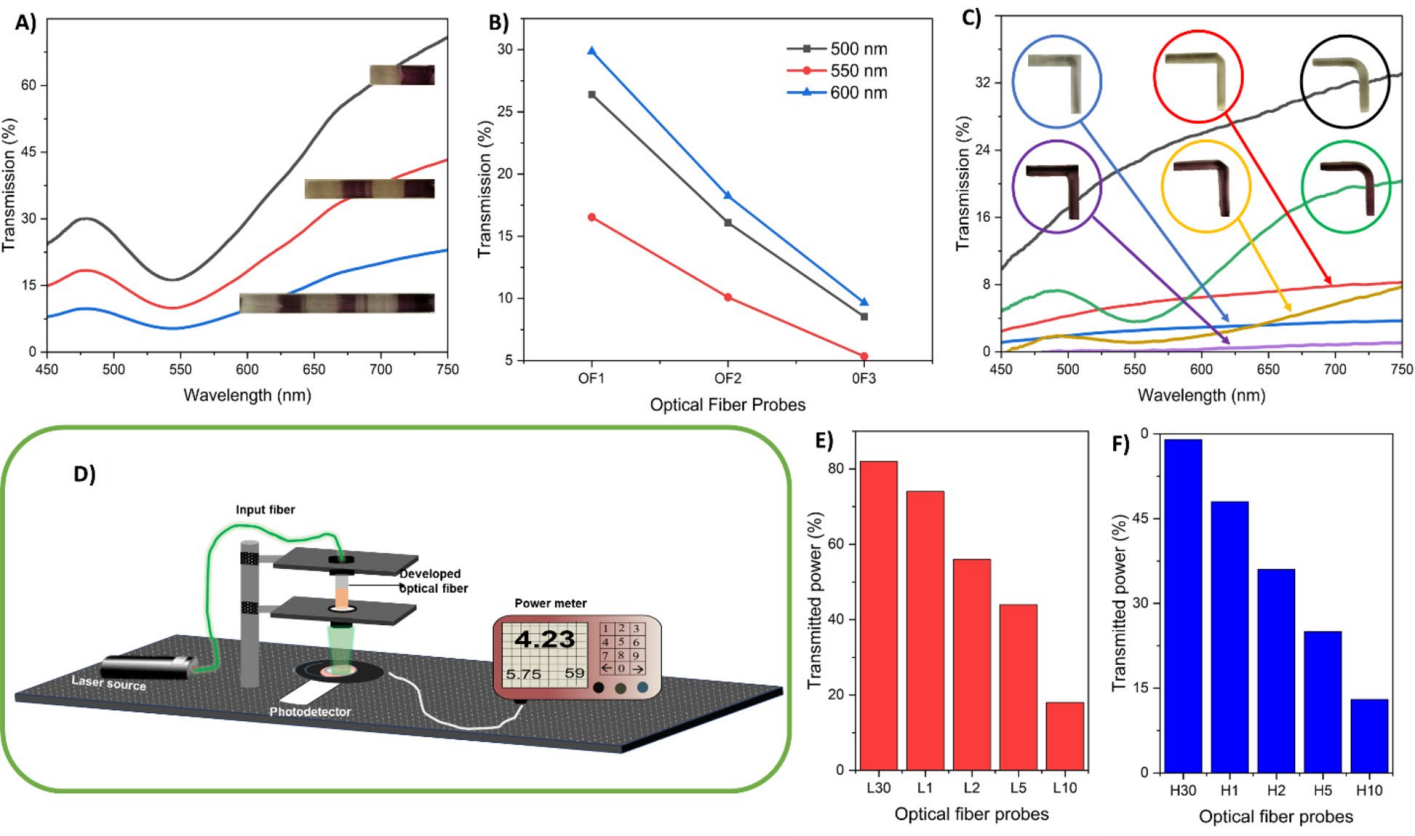
(**A**) Transmission spectra of multimaterial OFPs having different lengths (3 mm diameter, lengths 1 cm, 2 cm, and 3 cm), (**B**) transmission at 500, 550, and 600 nm (extracted from Fig. 5A), (**C**) transmission spectra of bends with AuNPs, (**D**) transmission measurement setup using laser, (**E**) transmitted power of OFP (low-AuNPs) using green laser, (**F**) and transmitted power of OFP (high-AuNPs) using green laser.

Figure 5.A illustrates the transmission spectra of multimaterial OFPs having different lengths (3 mm diameter, lengths 1 cm, 2 cm, and 3 cm). The multimaterial fibers have alternated sections of Dentaclear (no embedded AuNPs) and HEMA/PEGDA (with AuNPs), each having lengths of 5 mm. The transmission is reduced with increases in length due to an increase in the NP driven plasmonic absorption and scattering. The transmission of these fibers at different wavelengths 500 nm, 550 nm, and 600 nm is shown in Fig. 5.B. The plasmonic nanoparticle doped regions act as optical filters and can be used as wavelength selective optical fibers for applications like photonic circuits and communications. Some other fiber shapes were also printed with the single HEMA-PEGDA resin and then dipped in low-AuNP precursor for a time duration of 10 min, which is shown in Fig. 5.C. The waveguides having sharp 90 degrees bend transmit light very minimally compared to the waveguides with a 45^0^ tapered edge and round edges. The introduction of nanoparticles reduces overall transmission and there was a dip in the spectra in the 500 to 600 nm region due to the LSPR. Figure 5.D illustrates the transmission measurement setup of OFPs using a green laser with a wavelength of 532 nm. Instead of a UV spectrophotometer, a power meter is used to monitor the transmitted power through the fiber. Figure 5.E and Fig. 5.F depicts the transmitted power via the OFPs dipped in low-AuNP solution and high-AuNP solution, respectively. The transmitted power of OFP (low-AuNPs) is higher than that of OFP (high-AuNPs) of the 532 nm green laser, which underlines the correctness of the transmission results obtained from the visible light. The transmitted powers of OFP (low AuNPs) are 82%, 74%, 56%, 44%, and 18% for the dipping times of 30 s, 1 min, 2 min, 5 min, and 10 min, respectively. Similarly, for OFP (high AuNPs), the transmission is 59%, 48%, 36%, 25%, and 13% for dipping times of 30 s, 1 min, 2 min, 5 min, and 10 min, respectively. The results demonstrate that 3D printing is a promising platform for printing transparent multimaterial waveguides in various geometries. And by using the novel reduction by polymer (HEMA) method demonstrated, plasmonic gold nanoparticles can be integrated at designated regions of the 3D printed optical fibers, for applications like wavelength selective filtering or broadband absorbance.

**Fig. 6:**
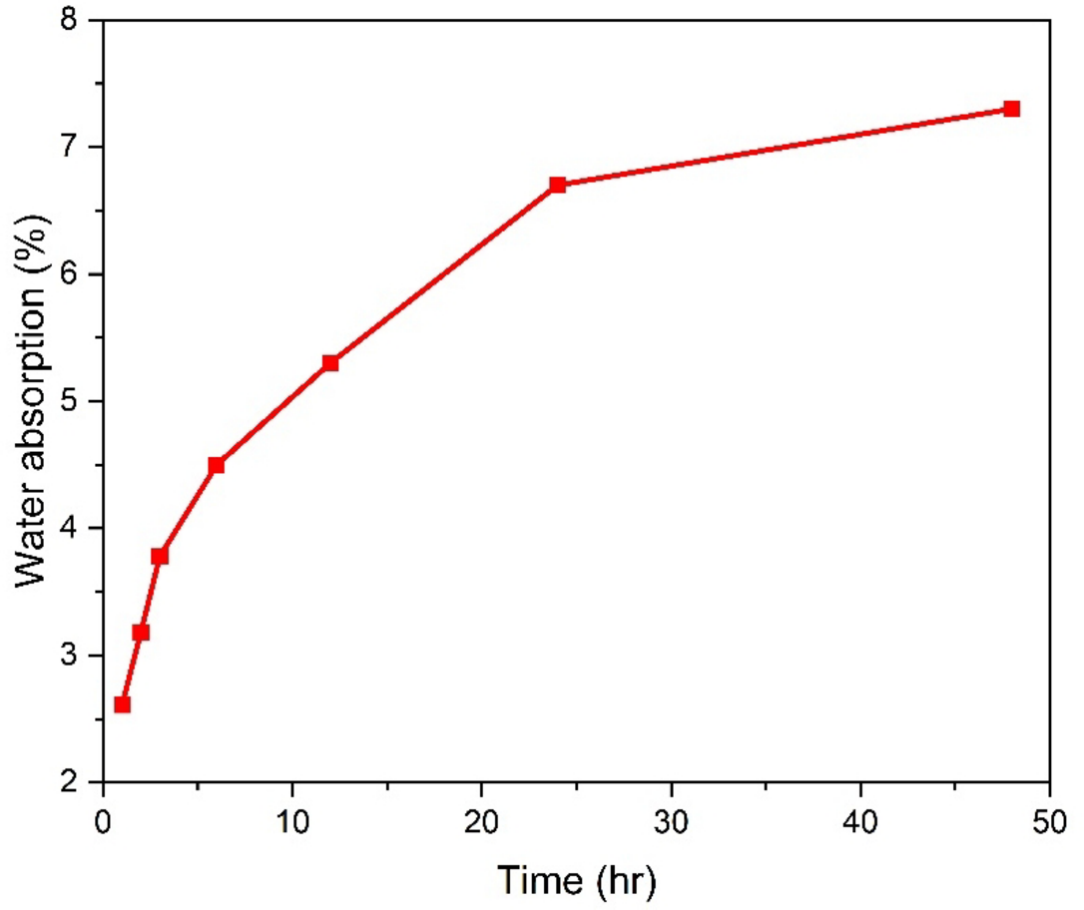
Water absorption results of L10-OFP at various time intervals.

Figure 6 shows the water absorption results of L10-OFP at various time intervals. The percentage of water absorption is computed using the formula:

$$\:\%\:of\:water\:absorption=\:\frac{Wf-Wi}{Wi}\times\:100$$

Before immersion in water, the samples are subjected to drying in an oven at 60 °C for one hour. The water absorption of the OFPs is 2.61%, 3.18%, 3.75%, 4.5%, 5.3%, 6.7%, and 7.3% during time intervals of 1, 2, 3, 6, 12, 24, and 48 h, respectively. No significant bending of the fibers was observed.

## Conclusions

In conclusion, using a polymeric reduction method, this study successfully demonstrated the in-situ synthesis of AuNPs within 3D-printed OFPs. A distinct LSPR in the 500–600 nm wavelength range, indicating the unique plasmonic properties imparted by incorporating AuNPs into the hydrogel polymer matrix of the OFPs, is observed. By varying the dipping time in the gold precursor solution, we could precisely control the concentration of AuNPs within the OFPs, allowing for tailored optical properties. The comprehensive characterization of the AuNP-loaded OFPs revealed their tunable light absorption, reflection, and transmission characteristics. Increased plasmonic absorption, resulting from higher AuNP concentrations and longer dipping times, reduced the amount of light passing through and reflecting. Furthermore, the fabricated OFPs demonstrated remarkable stability in their optical performance across varying temperature and pH conditions, underscoring their robustness and potential for diverse applications. These results pave the way for the development of improved optical devices, filters, and biomedical probes that harness the unique plasmonic properties of AuNPs integrated into 3D-printed polymer structures.

## Data Availability

The data used to support the findings are included in the article.

## References

[1] Tai, J., Fan, S., Ding, S. & Ren, L. Gold nanoparticles based optical biosensors for cancer biomarker proteins: a review of the current practices. *Front. Bioeng. Biotechnol.* **10**, 877193 (2022). 35557858 10.3389/fbioe.2022.877193 PMC9089302

[2] Sarfraz, N. & Khan, I. Plasmonic gold nanoparticles (AuNPs): properties, synthesis and their advanced energy, environmental and biomedical applications. *Chemistry–An Asian J.* **16**, 720–742 (2021). 10.1002/asia.202001202 33440045

[3] Salih, A. E., Elsherif, M., Alam, F., Yetisen, A. K. & Butt, H. Gold nanocomposite contact lenses for color blindness management. *ACS Nano* **15**, 4870–4880 (2021). 33570901 10.1021/acsnano.0c09657 PMC8023801

[4] Salih, A. E. et al. Syntheses of gold and silver nanocomposite contact lenses via chemical volumetric modulation of hydrogels. *ACS Biomater. Sci. Eng.* **8**, 2111–2120 (2022). 35468279 10.1021/acsbiomaterials.2c00174 PMC9092337

[5] Guo, Z. et al. Intrinsic optical properties and emerging applications of gold nanostructures. *Adv. Mater.* **35**, 2206700 (2023). 10.1002/adma.202206700 36620937

[6] Kim, S. & Yoon, S. On the origin of the plasmonic properties of gold nanoparticles. *Bull. Korean Chem. Soc.* **42**, 1058–1065 (2021).

[7] Khurana, K. & Jaggi, N. Localized surface plasmonic properties of au and ag nanoparticles for sensors: a review. *Plasmonics* **16**, 981–999 (2021).

[8] Zhao, J., Xue, S., Ji, R., Li, B. & Li, J. Localized surface plasmon resonance for enhanced electrocatalysis. *Chem. Soc. Rev.* **50**, 12070–12097 (2021). 34533143 10.1039/d1cs00237f

[9] Farkhani, S. M. et al. Tailoring gold Nanocluster Properties for Biomedical Applications: from sensing to Bioimaging and Theranostics. *Prog Mater. Sci.* 101229. (2023).

[10] Ghobashy, M. M., Alkhursani, S. A., Alqahtani, H. A., El-damhougy, T. K. & Madani, M. Gold nanoparticles in microelectronics advancements and biomedical applications. *Mater. Sci. Engineering: B* **301**, 117191 (2024).

[11] Hassan, H. et al. Gold nanomaterials–the golden approach from synthesis to applications. *Mater. Sci. Energy Technol.* **5**, 375–390 (2022).

[12] Park, W. et al. Advanced hybrid nanomaterials for biomedical applications. *Prog Mater. Sci.* **114**, 100686 (2020).

[13] Abhishek, N., Verma, A., Singh, A. & Kumar, T. Metal-conducting polymer hybrid composites: a promising platform for electrochemical sensing. *Inorg. Chem. Commun.* 111334. (2023).

[14] Sagadevan, S. et al. Recent advancements in polymer matrix nanocomposites for bone tissue engineering applications. *J. Drug Deliv Sci. Technol.* **82**, 104313 (2023).

[15] Ismail, R. A., Almashhadani, N. J. & Sadik, R. H. Preparation and properties of polystyrene incorporated with gold and silver nanoparticles for optoelectronic applications. *Appl. Nanosci.* **7**, 109–116 (2017).

[16] Muranaka, K., Niidome, T., Torres-Mapa, M. L., Heisterkamp, A. & Terakawa, M. Formation of gold nanoparticles inside a hydrogel by Multiphoton Photoreduction for Plasmonic Sensing. *Plasmonics* **18**, 751–760 (2023).

[17] Yassin, A. Y. Synthesized polymeric nanocomposites with enhanced optical and electrical properties based on gold nanoparticles for optoelectronic applications. *J. Mater. Sci.: Mater. Electron.* **34**, 46 (2023).

[18] Al-Shamari, A. A., Abdelghany, A. M., Alnattar, H. & Oraby, A. H. Structural and optical properties of PEO/CMC polymer blend modified with gold nanoparticles synthesized by laser ablation in water. *J. Mater. Res. Technol.* **12**, 1597–1605 (2021).

[19] Wang, L., Hasanzadeh Kafshgari, M. & Meunier, M. Optical properties and applications of plasmonic-metal nanoparticles. *Adv. Funct. Mater.* **30**, 2005400 (2020).

[20] Philip, A. & Kumar, A. R. The performance enhancement of surface plasmon resonance optical sensors using nanomaterials: a review. *Coord. Chem. Rev.* **458**, 214424 (2022).

[21] Di Cianni, W. et al. Polymer nanocomposites for plasmonics: in situ synthesis of gold nanoparticles after additive manufacturing. *Polym. Test.* **117**, 107869 (2023).

[22] Schuster, K. et al. Material and technology trends in fiber optics. *Adv. Opt. Technol.* **3**, 447–468 (2014).

[23] Tong Ph, X. C., D, X. C. & Tong *Opt. Fibers Adv. Mater. Integr. Opt. Waveguides* 161–211. (2014).

[24] Zhang, X. & Liou, F. *Introduction to Additive Manufacturing*pp. 1–31 (in: Addit Manuf, Elsevier, 2021).

[25] Awari, G. K., Thorat, C. S., Ambade, V. & Kothari, D. P. *Additive Manufacturing and 3D Printing Technology: Principles and Applications* (CRC, 2021).

[26] Gao, H. et al. 3D printed optics and photonics: processes, materials and applications. *Mater. Today* **69**, 107–132 (2023).

[27] Ali, M., Alam, F. & Vahdati, N. Butt, 3D-Printed holographic fresnel lenses. *Adv. Eng. Mater.* **24**, 2101641 (2022).

[28] Alam, F., Ali, M., Elsherif, M., Salih, A. E. & El-Atab, N. Butt, 3D printed intraocular lens for managing the color blindness. *Additive Manuf. Lett.* **5**, 100129 (2023).

[29] Dileep, C., Jacob, L., Umer, R. & Butt, H. Review of Vat photopolymerization 3D Printing of Photonic Devices, Addit Manuf 104189. (2024).

[30] Han, D. & Lee, H. Recent advances in multi-material additive manufacturing: methods and applications. *Curr. Opin. Chem. Eng.* **28**, 158–166 (2020).

[31] An, J. & Leong, K. F. Multi-material and multi-dimensional 3D printing for biomedical materials and devices. *Biomedical Mater. Devices* **1**, 38–48 (2023).

[32] Nazir, A. et al. Multi-material additive manufacturing: a systematic review of design, properties, applications, challenges, and 3D printing of materials and cellular metamaterials. *Mater. Des.* **226**, 111661 (2023).

[33] Alam, F., Elsherif, M., Salih, A. E. & Butt, H. 3D printed polymer composite optical fiber for sensing applications. *Addit. Manuf.* **58**, 102996 (2022).

[34] Daminabo, S. C., Goel, S., Grammatikos, S. A., Nezhad, H. Y. & Thakur, V. K. Fused deposition modeling-based additive manufacturing (3D printing): techniques for polymer material systems. *Mater. Today Chem.* **16**, 100248 (2020).

[35] Shaukat, U., Rossegger, E. & Schlögl, S. A review of multi-material 3D printing of functional materials via vat photopolymerization. *Polym. (Basel)* **14**, 2449 (2022). 10.3390/polym14122449 PMC9227803 35746024

[36] Vallejo Melgarejo, L. D., García, J., Reifenberger, R. G. & Newell, B. Manufacture of lenses and diffraction gratings using DLP as an additive manufacturing technology, in: Smart Materials, Adaptive Structures and Intelligent Systems, American Society of Mechanical Engineers, : p. V002T08A004. (2018).

[37] Dumitrescu, G. D. et al. Development of new hybrid casein-loaded PHEMA-PEGDA hydrogels with enhanced mineralisation potential. *Materials* **15**, 840 (2022). 35160786 10.3390/ma15030840 PMC8836935

[38] Farzanfar, J., Farjadian, F., Roointan, A., Mohammadi-Samani, S. & Tayebi, L. Assessment of pH responsive delivery of methotrexate based on PHEMA-st-PEG-DA nanohydrogels. *Macromol. Res.* **29**, 54–61 (2021).

[39] Eren, T. N., Lalevee, J. & Avci, D. Water soluble polymeric photoinitiator for dual-curing of acrylates and methacrylates. *J. Photochem. Photobiol Chem.* **389**, 112288 (2020).

